# Increased cell size and cadherin 11 expression following metabolic adaptation in SCC-25 oral squamous carcinoma cells

**DOI:** 10.1016/j.bbrep.2026.102869

**Published:** 2026-11-10

**Authors:** Jennifer C. Jones, Amy E. Hensel, Maria Cuevas-Nunez, Thomas M. Bodenstine

**Affiliations:** a Department of Biochemistry & Molecular Genetics, Midwestern University, 555 31st Street, Downers Grove, IL, 60515, United States; b College of Dental Medicine, Midwestern University, 555 31st Street, Downers Grove, IL, 60515, United States; c College of Graduate Studies, Midwestern University, 555 31st Street, Downers Grove, IL, 60515, United States

**Keywords:** Cadherin 11, Metabolism, Oral squamous cell carcinoma

## Abstract

Cadherin 11 (CDH11) is a transmembrane cell adhesion protein with roles in oral biology and development. Increased expression of CDH11 has been correlated with aggressive disease in multiple cancer types including those of the head and neck region. Specifically, oral squamous cell carcinoma (OSCC) is an aggressive malignancy prone to invasion and metastasis and associated with poor outcomes and therapeutic resistance. Cellular qualities of OSCC vary during tumor growth in response to changes in blood perfusion and nutrient availability. The resulting metabolic stress promotes reprogramming responses that lead to aggressive cancer cell qualities. Alterations to CDH11 dynamics in response to nutrient limitations in the context of oral cancer have not been well characterized. The aim of this study was to evaluate changes to CDH11 expression and localization following metabolic adaptation of the SCC-25 OSCC cell line when exposed to reduced glucose availability. Adapted cells exhibited high viability with an increase in average cell size. CDH11 expression in this population increased at both the transcript and protein levels. Additionally, CDH11 demonstrated greater localization to the plasma membrane at cell-cell junctions following adaptation. The increased expression and relocalization under these conditions suggests a potential role for CDH11 in response to nutrient stress, providing rationale for more comprehensive functional and molecular investigation.

## Highlights

• Characterization of CDH11 in SCC-25 cells
• Dynamic changes to CDH11 in response to metabolic adaptation
• Evidence for potential selection and adaptation responses
• CDH11 connections to cellular metabolism

## 1 Introduction

Oral squamous cell carcinoma (OSCC) is a malignancy of the head and neck region [1]. OSCC tumors frequently progress to local invasion and subsequent metastasis, with advanced stage leading to decreased 5-year survival rates [1], [2]. Surgery and radiation are mainstays of treatment in early disease but can be challenging due to the anatomical location of the tumor. These approaches frequently result in complications regarding pain, deglutition, speech and appearance, which reduce overall quality of life and limit therapeutic application [3], [4]. Recurrent and advanced disease are often treated with combinatorial chemotherapy and immune-checkpoint inhibition, however OSCC cells frequently exhibit therapeutic resistance and tumor recurrence [5], [6], [7], [8], [9], [10]. Moreover, heterogeneity of tumor cells and changes to cancer cell metabolism during tumor progression contribute to the emergence of aggressive populations [11], [12]. These effects are facilitated by tumor hypoxia and variability in blood perfusion which limit oxygen and nutrients [11], [13], [14]. In line with these observations, studies have demonstrated that metabolic profiles of oral cancers evolve during tumor growth [15], [16], [17], [18].

Cadherin 11 (CDH11) is a Ca^2+^ dependent transmembrane attachment protein and member of the cadherin family important for epithelial cell function and involved in numerous processes of oral and craniofacial development [19], [20]. CDH11 predominantly forms homophilic interactions between cells and links to the actin cytoskeleton through a complex with α and β-catenin proteins. Through these interactions, CDH11 transmits mechanical input that modifies multiple signal transduction pathways leading to cellular effects on shape, motility and growth [21]. These roles demonstrate the potential for CDH11 to modulate qualities that affect cellular growth and survival. CDH11 also mediates interactions with the extracellular matrix through associations with molecules such as syndecan-4 and integrins where it plays a role in cellular motility [22], [23], [24]. Increased CDH11 expression has been implicated in advanced forms of cancer [21]. More specifically, numerous studies have linked CDH11 to increased invasion and metastasis while other reports have demonstrated tumor suppressive qualities, indicating context dependent effects which may be affected by a milieu of metabolic changes [25], [26], [27], [28], [29], [30], [31], [32].

In this study, we used the SCC-25 cell line to evaluate the expression of CDH11 following adaptation to growth conditions involving reduced glucose availability to loosely mimic a common metabolic pressure encountered by cancer cells during tumor growth. The SCC-25 cell line was originally derived from the tongue of a patient with a squamous cell carcinoma and has been utilized to characterize OSCC biology [33]. Given the data that support roles for CDH11 in advanced cancer, we explored CDH11 dynamics in regard to glucose availability to probe if CDH11 can be affected by nutrient stress in OSCC. Our data provide rationale for exploring the relationship of CDH11 in OSCC metabolic responses.

## 2 Materials and methods

### 2.1 Cell lines and authentication

The SCC-25 cell line was purchased from the American Type Culture Collection (ATCC) and cultured in RPMI 1640 (Sigma-Aldrich) supplemented with 10% fetal bovine serum (FBS) and 400 ng/ml hydrocortisone (complete media). Adapted populations were generated by culturing cells for 10 weeks using glucose-free RPMI 1640 supplemented with 10% FBS and 400 ng/ml hydrocortisone. FBS contains trace levels of glucose and this media was therefore subsequently referred to as reduced glucose media. Surviving cells were maintained in this growth media, exhibited high viability and considered an adapted population. Cells were grown in a humidified incubator at 37^°^C, 5% CO_2_ and passaged using trypsin-EDTA. Routine screening for *mycoplasma* species was performed via PCR detection using the Promokine PCR Mycoplasma Test Kit I/C. Cells were submitted to ATCC for cell line authentication during the time of experimentation and analyzed by short tandem repeat (STR) analysis, corresponding to a 100% match (17/17 STR alleles; Supplementary Fig. 1).

### 2.2 MTS assay

Cell viability was determined with MTS reagent using CellTiter 96 AQueous One Solution (Promega) according to manufacturer's protocol. Control cells were plated in triplicate in 96-well plates. 24 h after plating, two conditions were established involving continued growth in complete media described above or changed to reduced glucose media. Cells were grown for 6 days with media replaced at day 3. Results were quantified using a PerkinElmer EnSpire Plate Reader. Experiments were performed three independent times.

### 2.3 Scanning electron microscopy

Cells cultured on glass coverslips were fixed with 4% paraformaldehyde for 24 h at 4°C. After fixation, cells were washed with PBS and dehydrated at room temperature with the following increasing ethanol concentrations for 10 min each: 30%, 50%, 70%, 80%, 95%, 100%. Samples were dried in a Leica EM CPD300 critical point dryer and sputter coated on a Leica EM ACE600 with 15 nm silver. Imaging was performed on a JEOL JCM-6000Plus scanning electron microscope at 10 kV.

### 2.4 SYTOX AADvanced viability

A PBS wash and media change were performed on cells 24 h following plating to remove any floating cells and debris. Cells were allowed to grow undisturbed for 3 additional days before the assay was run. After 4 total days of incubation, the media and subsequent PBS wash were collected along with the trypsinized cells to ensure collection of all potentially detached dead cells. Invitrogen SYTOX AADvanced Dead Cell Stain was added to the mixture at a final concentration of 1 μM and incubated for 15 min at room temperature. Samples were analyzed on a Beckman Coulter CytoFLEX flow cytometer with CytExpert analysis software. SYTOX experiments were performed in triplicate wells three independent times.

### 2.5 Forward scatter cell size

Cells were grown to ∼50% confluence in triplicate plates before collection. Keeping cells subconfluent ensured that limited growth area was not a factor affecting cell morphology. Cells were dissociated with trypsin-EDTA, resuspended in 2% FBS/PBS, and analyzed on a Beckman Coulter CytoFLEX flow cytometer with CytExpert analysis software. Mean forward scatter area was used to compare cell size. Experiments were repeated three independent times.

### 2.6 Quantitative PCR

Following the manufacturers’ protocols, total RNA was extracted in triplicate from SCC-25 control and adapted cells using the RNeasy Plus Mini Kit (Qiagen) and cDNA was reverse transcribed with the QuantiTect Reverse Transcription Kit (Qiagen). Quantitative PCR was performed with TaqMan Fast Advanced Master Mix on an Applied Biosystems StepOnePlus Real-Time PCR system using CDH11 (assay ID: Hs00901479_m1) and TBP (assay ID: Hs00427620_m1) TaqMan primer probes. For analysis, *CDH11* expression was normalized to *TBP* expression (ΔCt), compared to the average *CDH11* control expression (ΔΔCt), and represented as a fold change (2^−ΔΔCt^).

### 2.7 Western blot

Three separate lysates were collected in lysis buffer containing 1% SDS, 5% glycerol and 25 mM Tris-HCl with protease and phosphatase inhibitors (Thermo Fisher Scientific). Lysates were passaged through a 21-gauge needle 12x, diluted in laemmli buffer with β-mercaptoethanol and heated at 95^°^C for 10 min. Protein concentrations were determined using BCA assay method. Lysates were resolved on a 12% SDS-polyacrylamide gel with 4% stacking and transferred to PVDF. Membranes were blocked in 5% non-fat dry milk in Tris-buffered saline with 0.1% tween-20 (TBST). CDH11 rabbit polyclonal (Cell Signaling Technology) and vinculin mouse monoclonal (Santa Cruz Biotechnology) antibodies were used for protein detection. Blots were developed with enhanced chemiluminescence reagent on a BioRad ChemiDOC MP imaging system. Densitometry was performed using NIH ImageJ software. CDH11 values of upper band shown in Fig. 3B were normalized to vinculin to calculate fold change across three independent experiments between control and adapted populations. Lower band detected in adapted cells may represent non-specific binding or processing forms of CDH11.

### 2.8 Immunofluorescence

Cells were grown on glass coverslips in 24-well plates and fixed in 100% cold methanol. Coverslips were mounted to glass slides using mounting media with DAPI (Sigma-Aldrich). Fixed cells were blocked in 2% BSA/PBS and incubated using CDH11 mouse monoclonal and β-actin rabbit monoclonal primary antibodies (Cell Signaling Technology) with detection using secondary AlexaFluor 488 and 594 antibodies (Thermo Fisher Scientific). Epifluorescence microscopy images were recorded using a Leica DMi8 microscope with LAS X software equipped with a DFC 9000 GT camera and an X-Cite XLED1 fluorescence source. Immunofluorescence experiments were performed using two coverslips per condition and performed three independent times.

### 2.9 Statistical analysis

GraphPad Prism 10 was used for statistical analysis. Student t-test was used to compare results between control and adapted cells. Significance was set at * p < 0.05 and **p < 0.01 as indicated in figure legends. Error bars represent standard deviation.

## 3 Results

We first evaluated the sensitivity of the SCC-25 cell line to growth in reduced glucose media. Cells were grown in complete or reduced glucose conditions for 6 days. Cells in complete media reached confluence by day 6 while culture in reduced glucose media was sufficient to induce cellular stress with morphologic changes apparent by phase microscopy (Fig. 1A). Viability measured using MTS reagent was significantly decreased in reduced glucose media compared to control (1.12 ± 0.09 to 0.64 ± 0.06; *p* < 0.01) (Fig. 1B).

**Fig. 1:**
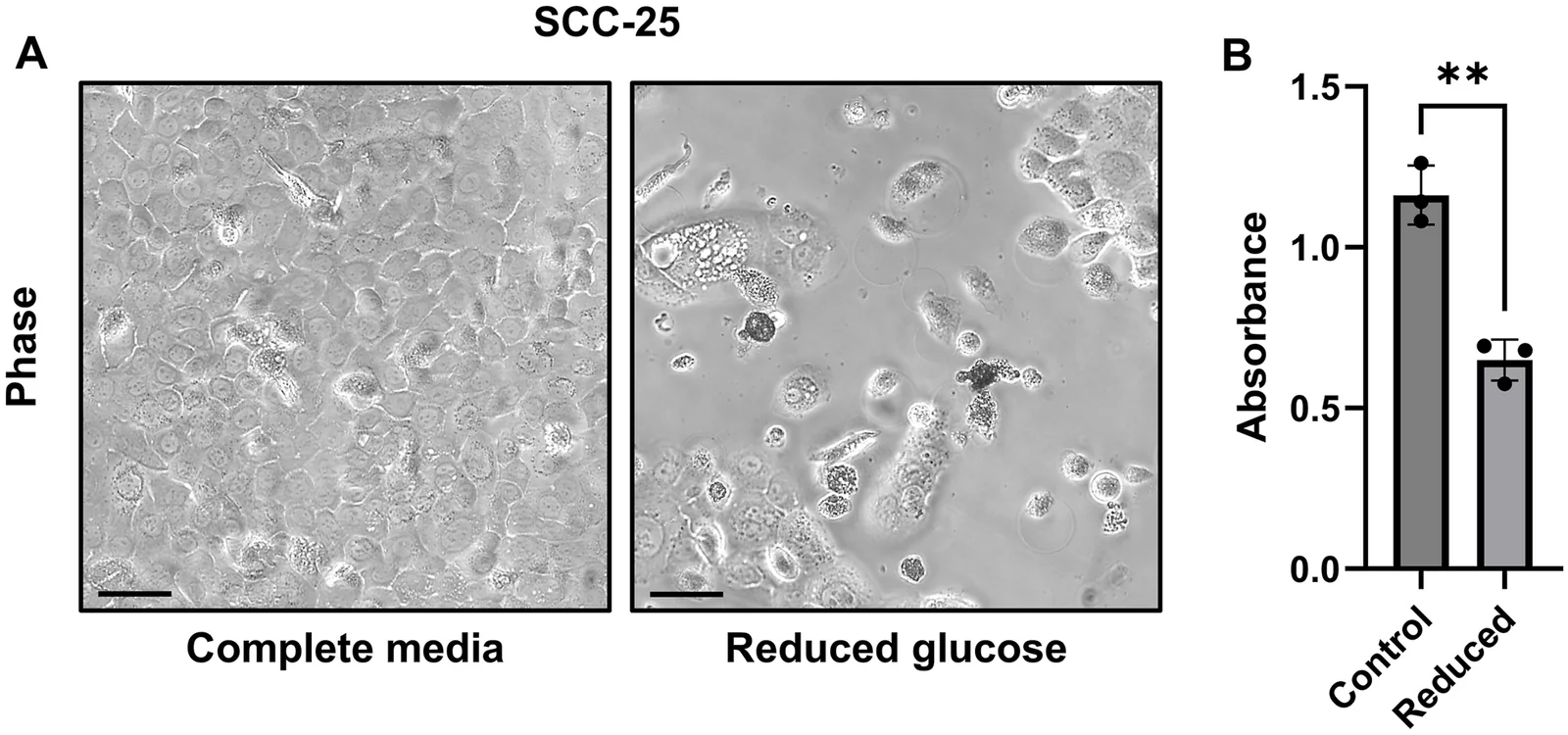
Glucose withdrawal reduces SCC-25 viability. (A) Representative phase microscopy of SCC-25 cultured for 6 days in complete or reduced glucose media; scale bar: 50 μm. (B) MTS viability assay showing absorbance measured from cells at 6 days of culture. Combined data of three independent experiments, **p < 0.01, ± SD.

To generate metabolically adapted SCC-25, growth in reduced glucose media was maintained for 10 weeks with media changes occurring every 2-3 days to remove dead cells and debris. Cells were passaged as necessary until a stable surviving population was established. This adapted population exhibited a variable increase in size that was noted by phase and scanning electron microscopy (Fig. 2A and B). To avoid the influence of potential changes to mitochondrial metabolism, SYTOX was used to assess viability between the cell populations. Control cells grown in complete media exhibited high viability at 92.3% ± 1.7%, while adapted cells exhibited a slight but significant decrease at 88.4% ± 0.3% (*p* < 0.05) (Fig. 2C). Changes to cell size were quantified by flow cytometry and exhibited a greater variability across the adapted population with an increase in forward scatter area (3.34 ± 0.13 to 4.78 ± 0.28 x10^6^; *p* < 0.01) indicating an overall increase in average size (Fig. 2D).

**Fig. 2:**
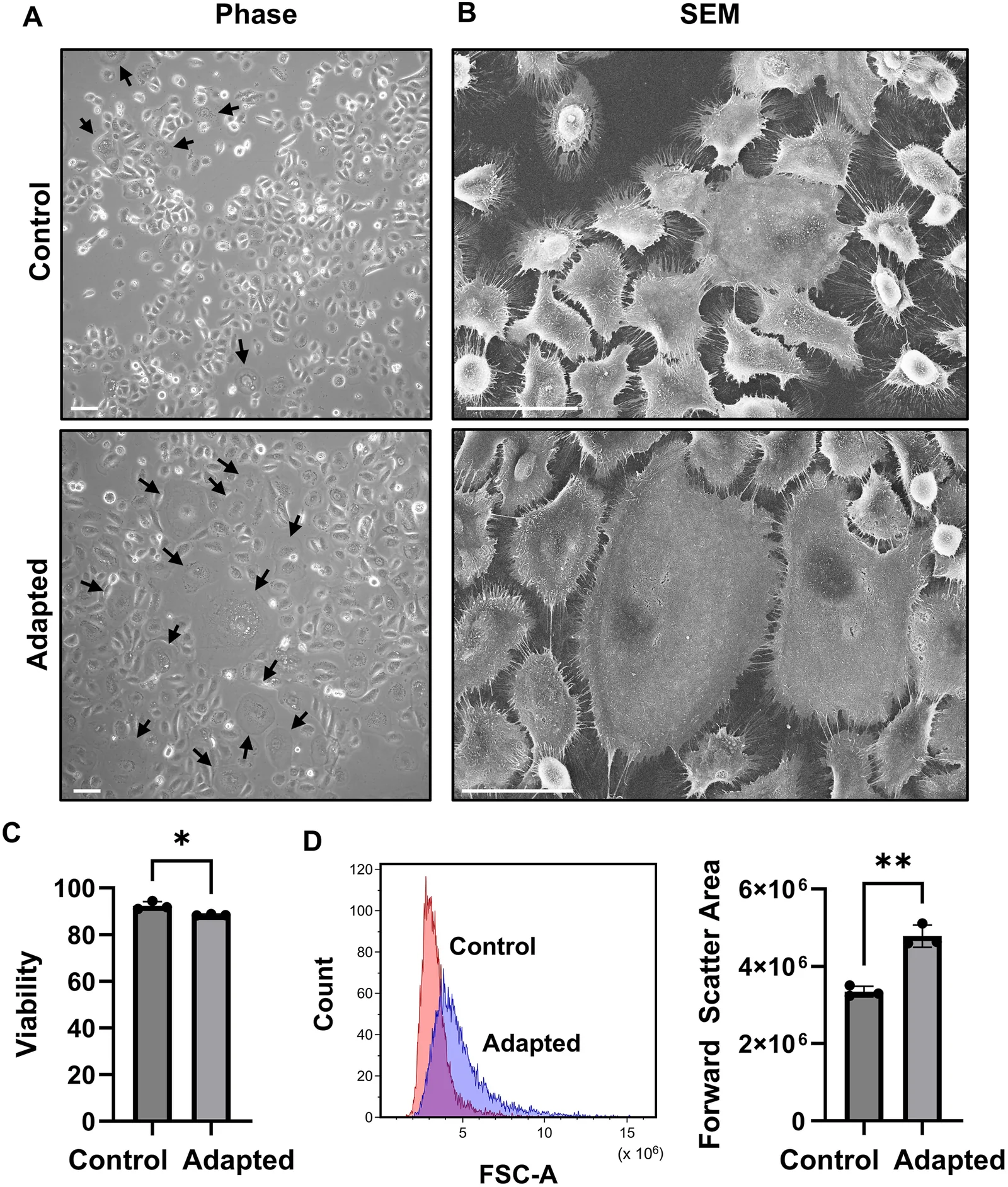
Adapted cells exhibit high viability with increased average cell size. (A) Phase microscopy of SCC-25 control and following metabolic adaptation; scale bar: 100 μm; arrows indicate large cells. (B) Scanning electron microscopy of control and adapted cells; scale bar: 50 μm. (C) SYTOX viability analysis of control and adapted populations. (D) Representative histogram analysis of flow cytometry on control and adapted cell populations correlating to cell size with quantification of average forward scatter area between each group. C and D represent combined data from three independent experiments. *p < 0.05, **p < 0.01, ± SD.

To evaluate changes in *CDH11* expression in the adapted population, we performed quantitative PCR and Western blot analysis. *CDH11* gene expression increased (4.14 ± 0.88 fold change; *p* < 0.01) in adapted SCC-25 cells and correlated to increased protein levels detected by Western blot analysis (2.93 ± 1.0 fold change; *p* < 0.01) (Fig. 3A and B). Immunofluorescence analysis was performed to examine potential changes to CDH11 localization between populations. Control cells exhibited less overall CDH11 fluorescence compared to adapted populations, in agreement with quantitative PCR and Western blot results (Fig. 3C). Control cell expression was heterogenous with the majority of cells exhibiting little to no detectable fluorescence, while a small fraction of cells displayed a non-membrane, vesicular pattern for CDH11 (Fig. 3C). In contrast, we observed increased overall expression of CDH11 in adapted cells, which was predominantly detected at cell-cell junctions. Membrane localized CDH11 in the adapted population did not appear to be dependent on cell size or confluence (additional fields shown in Supplementary Fig. 2). Interestingly, return of adapted cells to complete media demonstrated a progressive reversal of the effects on CDH11 protein levels from 8 to 10 weeks with a more consistent cellular morphology (Supplementary Fig. 3).

**Fig. 3:**
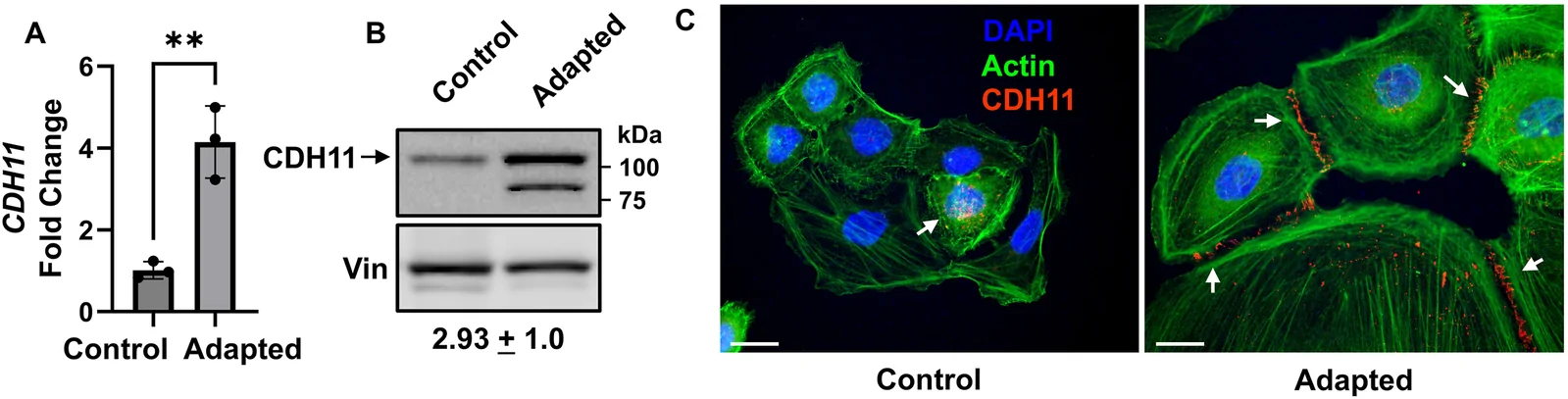
CDH11 expression and membrane localization increase following metabolic adaptation. (A) Quantitative PCR showing fold change in *CDH11* gene expression following adaptation to reduced glucose availability in SCC-25 cells. Combined results of three independent experiments, **p < 0.01, ± SD. (B) Representative Western blot analysis of CDH11 in control and adapted cells. Vinculin used as an equal loading control. Densitometry represents fold change results of three independent experiments ± SD. (C) Representative immunofluorescence images of CDH11 localization in control and adapted cells from three independent experiments. Blue: DAPI, green: β-actin, red: CDH11; scale bar: 20 μm. Arrows indicate CDH11. Additional fields of adapted cells shown in Supplementary Fig. 2.

## 4 Discussion

Metabolic adaptation during tumor development contributes to the emergence of cancer cell populations with increased survivability and motile phenotypes. While CDH11 plays physiologic roles in oral and craniofacial biology, it is upregulated in many oral malignancies and correlated with poor prognosis. As a class II cadherin family member, its adhesive properties are more transient, tissue specific, heterophilic, and overall, more indicative of migrating cells compared to class I family members such as E-cadherin [34]. Changes to glucose metabolism occur early during OSCC progression in response to tumor growth, competition for metabolites, hypoxia and nutrient depletion [35], [36]. Additional evidence has expanded upon mechanisms for how OSCC tumors direct glycolytic metabolism and induce metabolic flexibility when glucose is limited [37], [38]. A role for CDH11 in these processes may be linked to its influence on the β-catenin/WNT signaling pathway and its involvement in metabolic adaptation to stress [39], [40]. Moreover, a recent study revealed the ability of CDH11 to influence mitochondrial energy metabolism in bladder cancer, and we have previously demonstrated increased CDH11 expression in response to glucose withdrawal in breast cancer cells [41], [42]. Collectively, these findings suggest a potential role for CDH11 in response to nutrient stress. To explore this further, we induced a selective nutrient pressure in vitro using reduced glucose availability to determine the effects on expression of CDH11 in adapted populations of SCC-25 cells.

The population of stable, metabolically adapted SCC-25 cells generated in our study exhibited high viability and a wider range in cell size, with some cells becoming multiple times larger than control populations. Changes to cell volume occur in response to cellular stress and during cancer progression and correlate with our previous observations using similar conditions [42], [43], [44]. As metabolic plasticity provides cells with increased survivability, the increase in CDH11 at both the transcript and protein levels we observed support its reported associations to invasive qualities and correlation with poor prognosis in OSCC [27], [29], [45]. The significance of CDH11 in tumor progression is related in part to its connections with the cytoskeleton and effects on cellular motility. CDH11 has been shown to facilitate epithelial-to-mesenchymal transition, alter migratory pathways and affect the extracellular tumor environment [21], [23], [46], [47], [48], [49], [50]. Its upregulation in response to metabolic stress may direct cancer cell movement to regions of higher nutrient availability through its transient interactions and associations with focal adhesions, ultimately promoting survival and increasing metastatic potential.

Expression of CDH11 has not been extensively studied in the SCC-25 cell line. Our immunofluorescence results suggest a potential subpopulation of CDH11 expressing cells that could represent a population selected under our culture conditions. However, in the adapted population, CDH11 levels not only increased, but underwent overt relocalization to the plasma membrane at cell junctions. These observations suggest potential for both selection as well as adaptation responses, and an inherent ability to survive growth limiting conditions. The existence of heterogeneity and subpopulations within commercially available cell lines is well established, including within the SCC cell line series [51], [52], [53], [54], [55]. In vivo, populations of cells with cancer stem cell-like qualities create a hierarchy that represent similar observations regarding tumor subpopulations [56], [57], [58]. Moreover, drug treatments and selective pressures can induce the emergence of reversible cell line populations in cell culture [59], [60]. Consistent with this, treatment of the SCC-25 cell line with 5-fluorouracil has demonstrated the selection of chemoresistant populations [61]. The extended growth in adaptation media to generate the cells used in our study may also represent clonal selection independent of preexisting stem cell-like populations, rather than a concerted adaptation response. Collectively, further analysis is necessary to evaluate subpopulations within oral squamous cell carcinoma cell lines.

Limitations of our study include the lack of a mechanistic role for CDH11 in the adaptation response. Additionally, our work addresses glucose as a singular metabolic parameter, while the in vivo setting influences tumor evolution through a multitude of changes related to fluctuating fuel sources, oxygen limitation, and stromal effects. However, our in vitro data indicate an underlying connection that supports a broader potential role for CDH11 in metabolic plasticity. More specific exploration of CDH11 in adaptation and reprogramming responses are necessary further steps and will require detailed metabolomics approaches.

## CRediT authorship contribution statement

**Jennifer C. Jones:** Writing – review & editing, Validation, Methodology, Investigation, Formal analysis. **Amy E. Hensel:** Writing – review & editing, Investigation, Conceptualization. **Maria Cuevas-Nunez:** Writing – review & editing, Resources. **Thomas M. Bodenstine:** Writing – review & editing, Writing – original draft, Validation, Supervision, Resources, Project administration, Methodology, Investigation, Formal analysis, Conceptualization.

## Funding

This project was supported by the Midwestern University College of Graduate Studies and the College of Dental Medicine.

## Declaration of competing interest

The authors declare that they have no known competing financial interests or personal relationships that could have appeared to influence the work reported in this paper.

## Data Availability

Data will be made available on request.
