# Supplementary material for: Increased cell size and cadherin 11 expression following metabolic adaptation in SCC-25 oral squamous carcinoma cells
Source: Biochem Biophys Rep. 2026 Nov 10;48:102869. doi: 10.1016/j.bbrep.2026.102869 (PMC13770635; doi:10.1016/j.bbrep.2026.102869)
Supplement: Supplementary file 1 [file mmc1.pdf]

Supplementary Figures

	Submitted	Reference
% Match	100	100
ATCC® Cat. No.	STRC3640	CRL-1628
Designation	SCC-25	SCC-25; Tongue Carcinoma; Human (Homo sapiens)
D3S1358	17	17
TH01	8	8
D21S11	30	30
D18S51	16	16
D5S818	12	12
D13S317	13	13
D7S820	12	12
D16S539	11, 12	11, 12
CSF1PO	10	10
vWA	17, 19	17, 19
D8S1179	13	13
TPOX	8,12	8,12
FGA	20, 24	20, 24

Supp Fig 1. Validation of SCC-25 cell line. Short-tandem repeat analysis of SCC-25 cell line used in this study.

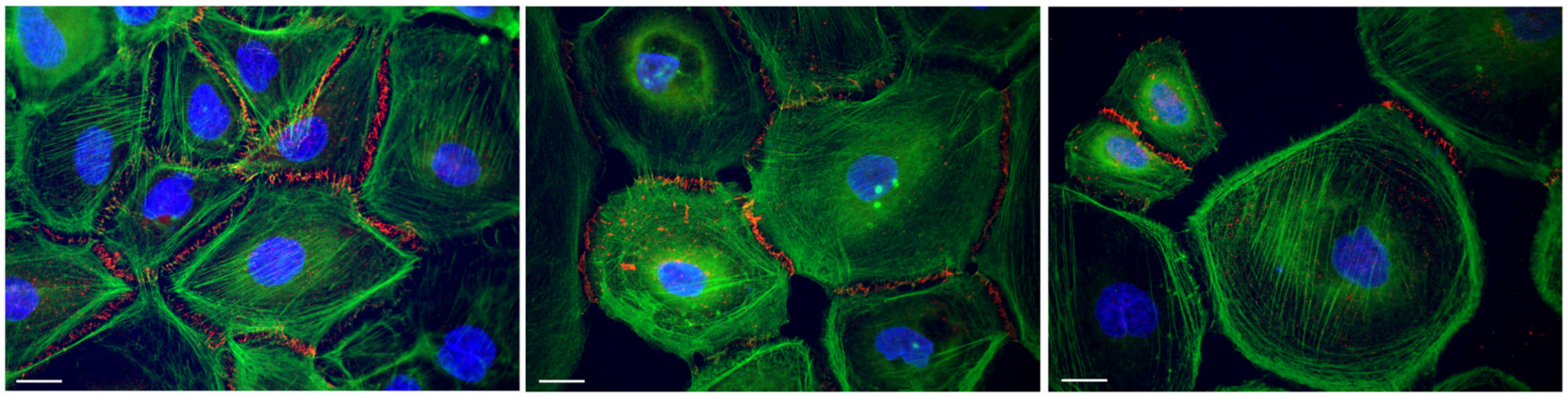

Supp Fig 2. CDH11 membrane localization in adapted populations. Additional fields (from Fig. 3C) of adapted SCC-25 cells exhibiting membrane localized CDH11. Blue: DAPI, green: β-actin, red: CDH11; scale bar: 20 μM.

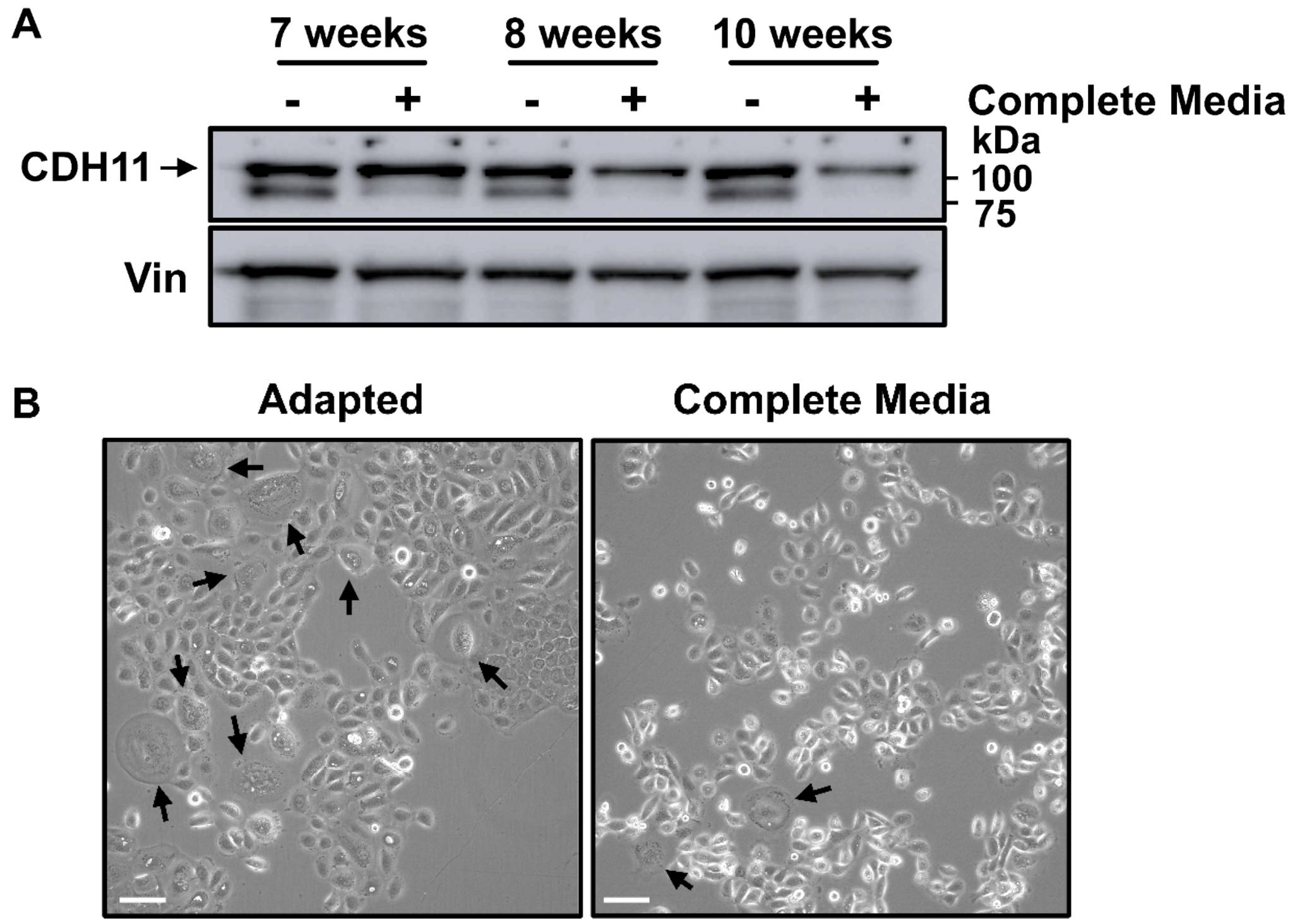

Supp Fig 3. Decreased CDH11 levels following return of adapted populations to normal growth media. (A) Western blot analysis of changes to CDH11 in adapted cells following growth in normal media for 7, 8 and 10 weeks. Vinculin used as an equal loading control. (B) Phase microscopy of adapted cells following growth in complete media for 10 weeks; scale bar: 100 µm; arrows indicate large cells.
